# Interactions Between Microplastics and Marine-Derived Polysaccharides: Binding Mechanisms and Bioavailability in Aquatic Systems

**DOI:** 10.3390/toxics13110928

**Published:** 2025-10-29

**Authors:** Marcin H. Kudzin, Martyna Gloc, Natalia Festinger-Gertner, Monika Sikora, Magdalena Olak-Kucharczyk

**Affiliations:** Łukasiewicz Research Network—Lodz Institute of Technology, Marii Sklodowskiej-Curie 19/27 Street, 90-570 Lodz, Poland

**Keywords:** adsorption, alginate, aquatic ecosystems, bioavailability, bioremediation, carrageenan, chitosan, marine polysaccharides, microplastics

## Abstract

Microplastics (MPs) are increasingly recognized as persistent pollutants in marine and freshwater systems. Their small size, widespread distribution, and ability to adsorb chemical contaminants raise concerns about ecological impacts and human exposure through aquatic food webs. In parallel, marine polysaccharides such as alginate, chitosan, and carrageenan have drawn interest as natural biopolymers with the capacity to interact with MPs. These interactions occur via electrostatic forces, hydrophobic effects, hydrogen bonding, and physical entrapment, influencing the fate and mobility of MPs in aquatic environments. This review critically examines the current state of knowledge on the binding mechanisms between MPs and marine-derived polysaccharides, emphasizing their role in modulating the transport, aggregation, and bioavailability of plastic particles. Recent efforts to modify these biopolymers for improved performance in sorption and stabilization applications are also discussed. Furthermore, analytical strategies for investigating MP–polysaccharide systems are outlined, and the practical limitations associated with scaling up these approaches are considered. The potential use of such materials in environmentally sustainable remediation technologies is explored, along with future research needs related to safety evaluation, lifecycle impact, and feasibility in real-world conditions.

## 1. Introduction

Until the early 20th century, the widespread use of synthetic polymers was limited to a few materials, notably Bakelite and celluloid. However, since the 1950s, global plastic production has grown exponentially—from approximately 2 million tonnes in 1950 to over 450 million tonnes annually today. Plastics have become integral to modern life due to their low cost, versatility, and hygienic properties, with applications spanning construction, consumer goods, healthcare, and food packaging [1]. As plastic use has surged, so too has concern over its environmental consequences [2,3,4]. Scientists estimate that over 8 million tons of plastic waste ends up in the oceans every year, and this amount will increase to 23–37 million metric tons per year by 2040 [5,6,7]. The largest area of microplastics has been recorded in seas and oceans, such as the Mediterranean Sea, but they are also present in inland waters, soil, air, food products and living organisms all over the Earth. The Mediterranean Sea has one of the largest concentrations of microplastics in the world due to its high population density and intensive shipping [8]. Global plastic production, primarily driven by major producers such as China, India, and the USA, is characterized by a continuous growth trend. Modifications with stabilizers, glass fibers, and waxes increase their resistance to abiotic factors and biodegradation. According to reports from international organizations related to ecology and environmental protection, only 14% of used plastics are recycled [9]. The term “microplastic” was introduced in 2004 by the authors of a study analyzing contamination of seabed sediments near Plymount, UK, but it referred only to general plastic particles. It was not until 2008, during the International Research Workshop on the Occurrence, Effects, and Fate of Microplastic Marine Debris, organized by the National Oceanic and Atmospheric Administration (NOAA) and the University of Washington, that the official terminology and definition of plastic particles of varying structure, size, and density were clarified [10]. Microplastics (MPs), defined as plastic fragments less than 5 mm in diameter, have emerged as a major pollutant in both marine and freshwater environments. These particles either originate from the breakdown of larger plastic waste (secondary MPs) or are manufactured intentionally in small sizes for use in cosmetics, textiles, and industry (primary MPs) [11,12,13,14,15,16]. It is predicted that the amount of microplastics in the oceans will continue to increase (certainly until 2066) [17]. Currently, the most common polymers found in the aquatic environment include polyethylene (PE), polypropylene (PP), polyester (PET), polystyrene (PS), polyvinyl chloride (PVC), and polyamide (PA) [18]. Owing to their durability and resistance to degradation, MPs can persist for extended periods in aquatic systems, accumulating in water columns, sediments, and organisms. A key concern regarding MPs lies in their ability to adsorb and transport hazardous substances, including persistent organic pollutants (POPs), heavy metals, and pharmaceutical residues [19,20,21,22]. These interactions can facilitate the transfer of contaminants through aquatic food webs, potentially affecting organisms from plankton to fish and eventually entering the human diet via seafood [23,24,25,26].

Recently, new methods for removing microplastics from water have begun to emerge, but adsorption using materials with a high affinity for capturing MPs is attracting considerable attention. Laboratory and pilot experiments have demonstrated the potential for microplastics to be collected by zeolites, activated carbon, functionalized polymers, and magnetic nanoparticles [27]. However, it should be noted that most adsorbents are not biodegradable and may contribute to secondary water pollution due to their durability and the chemicals used in their production [28]. Furthermore, the disposal and regeneration of used substances poses economic and logistical problems, particularly in large areas of seas and oceans [29]. Recent research has focused on the development of biodegradable adsorbents for the removal of MPs from saline waters. Particular attention is being paid to biosurfactants and natural polymers such as chitosan, alginate, cellulose, and polylactic acid, which offer natural biodegradability, environmental friendliness, potential scalability, and controllable surface chemistry [30].

Recent research has drawn attention to the role of natural marine biopolymers—particularly polysaccharides such as alginate, chitosan, and carrageenan—in influencing the environmental behavior of MPs. These biopolymers, derived from macroalgae and crustacean sources, are biodegradable, renewable, and rich in functional groups capable of interacting with plastic particles through electrostatic forces, hydrophobic associations, and hydrogen bonding [31,32]. Polysaccharides are a component of the marine snow commonly found in the oceans, which is an accumulation of organic and inorganic matter ranging in size from a few millimeters to even a few centimeters. Sea snow is formed from a variety of particles such as the remains of organisms, plankton faeces, minerals, bacterial mucus or dust carried from the atmosphere. Polysaccharides play a particularly important role in the structure of these aggregates, acting as binders to stabilize and strengthen their structure [33,34,35]. Sea snow not only participates in the circulation of matter in the oceans but also has an important function in the global transport of carbon to the deep sea—the so-called biological carbon pump. Thanks to this process, some organic carbon is permanently isolated in bottom sediments, which affects the Earth’s climate balance [33]. Given their favorable environmental profile and binding potential, marine polysaccharides have been explored as components of eco-friendly remediation strategies aimed at mitigating microplastic contamination. Chitosan, found in crab shells, and alginate, found in brown algae, are examples of polysaccharides with variable surface chemistry, enabling functional changes in nanoparticles (cross-linking or integration), which improves the adsorption capabilities of these polysaccharides. Studies have demonstrated their applicability in capturing, immobilizing, or even facilitating the degradation of MPs, positioning them as promising candidates in the development of sustainable technologies [30,36,37]. This review provides a detailed synthesis of current knowledge on the interactions between microplastics and marine-derived polysaccharides. It examines the underlying physicochemical mechanisms, evaluates their impact on the fate and bioavailability of MPs, and highlights recent advances in the use of polysaccharide-based materials for remediation. A better understanding of these processes may support the development of practical and environmentally sound approaches to address the global challenge of microplastic pollution.

## 2. Overview of Microplastic Pollution in Aquatic Environments

Microplastic pollution has become a critical environmental concern due to the widespread and persistent presence of microplastics (MPs) across diverse aquatic ecosystems globally. MPs have been detected in surface waters, water columns, sediments, and biota from coastal to open ocean environments, as well as in freshwater rivers and lakes, highlighting their pervasive distribution and potential to affect ecosystem functioning [24,38,39,40,41,42,43]. It is estimated that global leakage of plastic substances into the environment amounts to 10–40 million tons per year, and if we maintain the current trend, it is predicted that this amount will triple by 2040 [30]. Plastic waste affects or is consumed by more than 700 species of marine animals: fish, birds, and mammals, often causing health problems, reduced mobility, malnutrition, or death [44,45]. MPs and nanoplastics move into tissues and organs; recent studies have shown their presence in the digestive tracts of various marine organisms. Furthermore, MPs can affect animal behavior and reproduction, and can also act as toxic vectors of hazardous chemicals, including endocrine disruptors, through food webs [46,47].

**Figure 1:**
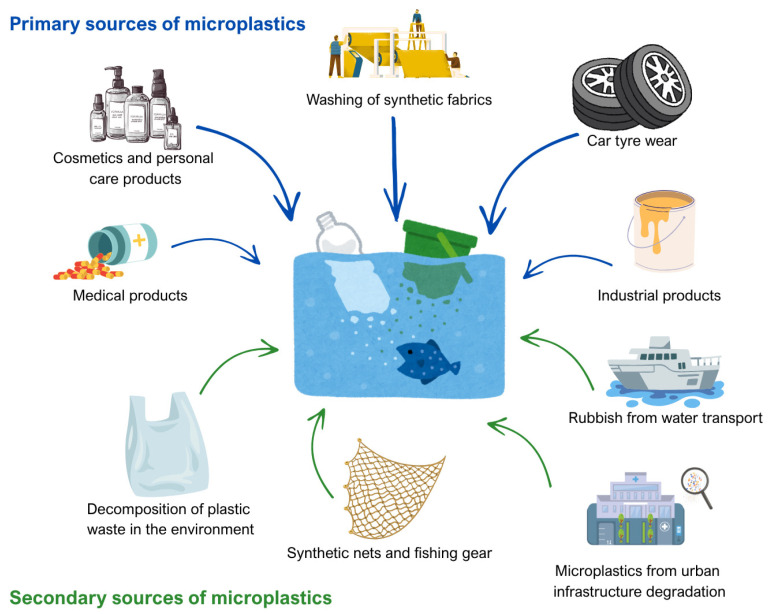
Sources of microplastics in the aquatic environment (figure prepared by the author).

The sources of MPs are multifaceted and include both primary microplastics, which are manufactured to be small for specific industrial and consumer uses, such as microbeads in cosmetics and synthetic fibers from textiles, and secondary microplastics, generated through the fragmentation of larger plastic debris due to physical, chemical, and biological weathering processes (Figure 1) [23,40,48]. The fragmentation of existing plastics continuously produces MPs, even if all new microplastic emissions were stopped. MPs are widespread and continue to accumulate. These particles have been found in over 1300 terrestrial and aquatic species, including organisms at the base of food webs and top predators. This shows how persistent these particles are in the environment and how dangerous they are [49].

**Figure 2:**
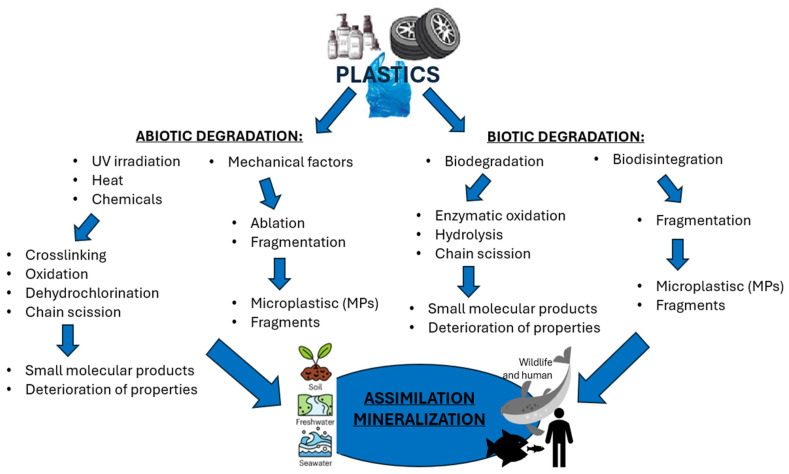
Decomposition of plastics in the environment (figure prepared by the author).

These particles vary widely in polymer composition, including polyethylene (PE), polypropylene (PP), polystyrene (PS), and polyvinyl chloride (PVC) (Table 1), each exhibiting distinct densities, morphologies, and degradation behaviors (Figure 2) [50,51]. The varying chemical and physical properties of polymers affect their behavior in the aquatic environment. Depending on these characteristics, plastics can float, drift in the water’s depths or sink to the bottom [52].

The shape, size, density and surface texture of the microplastic, and environmental conditions such as wind, waves, and UV radiation largely determine its transport in the environment, the extent of dispersion, bioavailability to organisms and the rate of adsorption of contaminants [30,53]. The small dimensions of microplastic particles allow them to be readily transported between environmental compartments and increase the likelihood of uptake by a wide range of aquatic organisms, from zooplankton and filter-feeding bivalves to fish and apex predators [31]. Their environmental persistence stems from a high resistance to biological degradation, resulting in prolonged accumulation and the potential for trophic transfer throughout aquatic food webs. MPs increase ecotoxicological risks because they act as vectors for pathogenic microorganisms and toxic compounds [54]. Moreover, MPs are known to adsorb and concentrate various pollutants—including persistent organic pollutants (POPs), heavy metals, and microbial pathogens—thus exacerbating ecological risks and raising concerns about human exposure through seafood consumption [16,26,55,56,57]. Literature data shows that the concentration of heavy metals on the surface of plastic particles can be up to 800 times higher than in the surrounding seawater [58]. Growing attention has been directed toward measuring MPs concentrations in different aquatic compartments and elucidating their primary sources, dispersal mechanisms, and environmental sinks. Among the most significant entry points into aquatic systems are wastewater treatment effluents, urban surface runoff, and maritime-related activities [59]. Once introduced, MPs can settle in sediments—particularly those composed of high-density polymers or particles bound to biofilms or organic matter—creating long-term exposure scenarios in benthic habitats [60,61,62]. Unfortunately, the presence of microplastics has also been observed in marine snow. Plastic particles embedded in these aggregates sink faster to the ocean floor, promoting the transfer of pollutants from the water surface to the deep, where they can negatively affect benthic life [33,63,64]. To effectively model MPs transport and fate, it is essential to understand their dynamic interactions with environmental parameters, including natural organic matter, microbial consortia, and local hydrodynamic conditions. These interactions influence the mobility, degradation, and bioavailability of MPs. Furthermore, the lack of standardized protocols for sampling, identification, and quantification continues to pose challenges for data comparability across regions and studies. Establishing harmonized methodologies is a key prerequisite for global monitoring and mitigation efforts [65,66].

## 3. Toxicity of Microplastics

Microplastics formed because of the decomposition of plastic objects enter water and sediments, gradually increasing their concentration. Aquatic organisms, including fish, crabs, shrimp, and shellfish, ingest microplastic particles along with water and food. These pollutants accumulate in living organisms to varying degrees [67]. MPs present in living organisms can affect eating disorders, causing weight loss, stunted growth, and fertility problems [68], but they can also be neurotoxic, cardiotoxic, and cause oxidative stress in brain tissue, behavioral disorders, damage to the mucosal epithelium, metabolic disorders, and changes in heart rate [67,69]. MPs are naturally transferred to organisms at higher trophic levels in the food chain. Therefore, they can be transferred to humans through the consumption of aquatic products [70].

Microplastics pose a threat to the health and life of many living organisms, both aquatic and terrestrial. Because microplastic particles are hydrophobic, they attract and retain various chemical pollutants, mainly heavy metals, persistent organic pollutants, and plastic additives. Humans are exposed to microplastics from the sea because pollutants accumulate after consumption of seafood (shrimp, salmon, mussels, or tuna). Chronic exposure of living organisms to pollutants is associated with the risk of endocrine disruption, birth defects, and increased susceptibility to many diseases [71]. MPs affect many physiological systems in humans. Inhaling particles in the air leads to respiratory diseases such as asthma and chronic obstructive pulmonary disease. Recent studies have also highlighted the link between microplastic consumption and gastrointestinal diseases. MPs mainly cause inflammation of the digestive system. What is more, MPs have the ability to migrate, for example, to the brain, causing inflammation and neurodegenerative diseases. They also have a negative impact on the functioning of the immune system by disrupting the immune response. This makes people less resistant to infections and autoimmune diseases. Researchers have also found that MPs are linked to cancer and cardiovascular disease, leading to heart attacks and strokes. Furthermore, microplastics affect reproductive health by reducing fertility. It follows that there is an urgent need for further research and the development of effective techniques for cleaning the environment of plastic particles in order to reduce the risk of diseases resulting from contact with them [72,73,74,75].

### Microplastic Accumulation in Human Organisms—Health Consequences

Inhalation, direct or indirect ingestion, and dermal contact are the main routes of MP entry into the human body. Approximately 39,000–52,000 particles, or approx. 0.2–6035 units g^−1^, is the estimated average amount of plastic particles consumed annually through food, including consumption of aquatic animals, contaminated crops and beverages, and dermal contact Via cosmetics. The most common polymers are PE, HDPE, PET, and PS, with critical amounts occurring in India [76,77]. Constant exposure of the body to MPs leads to their accumulation. Recent studies have shown that the primary site of MP accumulation is the gastrointestinal tract (mainly the small intestine) and lymph nodes, liver, and spleen [78]

The surface of MP attracts not only hydrophobic pollutants, toxins, and microorganisms but also certain carcinogens, including polycyclic aromatic hydrocarbons, consequently increasing their bioavailability and absorption [79]. The main consequences of contaminants and pathogens entering the human gastrointestinal tract are disruption of the intestinal mucosal barrier and inflammation. Persisting in this state leads to disorders such as intestinal dysbiosis (dysbacteriosis), a weakening of the intestinal microbiome, and leaky gut syndrome (leaky gut syndrome), a condition known as intestinal permeability. Recent research indicates a link between MP accumulation and carcinogenesis and the development of pathogenic bacteria in the body [80].

Once in the body, MPs can circulate through the bloodstream and reach organs, crossing primary tissue barriers. For example, carboxylated nanoparticles tested in vitro can penetrate and adsorb onto red blood cells through hydrogen bonds, hydrophobic, electrostatic, and van der Waals forces with polystyrene. MPs can reach secondary barriers, such as the placental barrier and the blood–brain barrier, through the bloodstream [81]. Using spectroscopic techniques such as Raman spectroscopy, the number of MPs identified in stool samples was estimated at approximately 28–41 pieces/g [82]. MPs were also detected in lung tissue in the form of microfibers, indicating that MPs could also enter the human body via inhalation. MP accumulation contributes to the induction of oxidative stress, pro-inflammatory reactions and toxicity. Nanoplastics (NP) are considered to be probably more dangerous because, due to their smaller size than MP, they can penetrate cells and migrate to tissues [83]. Initial studies (Tong et al.) using mouse models of MP exposure at biologically relevant doses demonstrate adverse changes in the gut microbiome, the development of pathogenic pathogens (primarily *Enterobacteriaceae* and *Desulfovibrionaceae*), and progressive inflammation of the small intestinal mucosa [84]. The Inflammatory pathway may proceed through the activation of pro-inflammatory cytokines: interleukin-6 (IL-6) and interleukin-8 (IL-8), and the activation of macrophages, neutrophils, and dendritic cells. These processes may be accompanied by increased production of reactive oxygen species (ROS), resulting in the induction of apoptosis, a crucial link in the process of carcinogenesis [85].

In 2019, the WHO published a report concluding that, based on current knowledge, microplastics pose no immediate risk to human health. However, the organization emphasized the need for further research to understand the impact of microplastics, particularly smaller particles that can be absorbed by the body and carry chemicals. The WHO identified three potential risks of exposure to microplastics: physical (organ damage), chemical (poisoning), and microbiological (transfer of microorganisms) [86].

## 4. Marine-Derived Polysaccharides: Properties and Applications

Marine biomacromolecules are large biological molecules, such as polysaccharides, proteins, and lipids, derived from marine organisms such as algae, fish, and shellfish. They include polysaccharides (e.g., alginate, chitosan, hyaluronic acid) and proteins (e.g., collagen) and have a variety of unique properties. They are mainly used in the biomedical and pharmaceutical industries and in the production of biomaterials due to their biocompatibility and bioactivity [87].

Marine-derived polysaccharides are high-molecular-weight carbohydrates extracted primarily from marine macroalgae and crustaceans. The main polysaccharides discussed here include alginate, chitosan, and carrageenan, each possessing distinct chemical structures and functional groups (Table 2) that contribute to their versatile physicochemical properties and environmental applications [88].

Alginate, a naturally occurring polysaccharide extracted primarily from brown algae (Phaeophyceae), is composed of β-D-mannuronic (M) and α-L-guluronic acid (G) residues arranged in homopolymeric (M/M or G/G) and heteropolymeric (M/G) blocks. This structural arrangement imparts alginate with the unique ability to form hydrogels in the presence of divalent cations, particularly calcium ions (Ca^2+^), which is widely exploited in encapsulation technologies and pollutant sorption systems. The abundance of carboxylate groups contributes to its anionic character, enabling electrostatic interactions with positively charged substances, including metal ions and polymeric pollutants [39,48,59,89,90]. In addition, thanks to its properties, alginate is used in the production of edible films (tasteless, odourless, food grade ingredient), in tissue engineering to immobilize cells and control drug release (potential of gelling, stabilizing, thickening, and biocompatibility), and as a scaffold in cultured meat production (edible, non-allergenic, biodegradable, palatable) [91,92].

Chitosan is obtained through the partial deacetylation of chitin, which is found in the exoskeletons of marine crustaceans. Structurally, it is a linear polysaccharide composed of glucosamine and N-acetylglucosamine units. In acidic media, chitosan becomes positively charged due to the protonation of its amino groups, making it highly effective in binding negatively charged molecules, including anionic dyes, organic acids, and microplastics. Its biodegradability, low toxicity, and high affinity for pollutants have facilitated its use in a range of fields including water purification, agriculture, drug delivery, and as a hydrogel used in wound healing dressings [60,61,62,63,93].

Carrageenan, sourced from various species of red algae (Rhodophyceae), is a sulfated polysaccharide composed predominantly of alternating galactose residues. It exists in several forms—κ (kappa), ι (iota), and λ (lambda)—which differ in the degree and position of sulfation. These structural variations influence not only their gel-forming capacity but also their potential for binding cationic compounds. The negatively charged sulfate groups present in carrageenan facilitate ionic interactions and the formation of complexes with metal ions and other environmental contaminants [65,94,95,96,97]. Thanks to its properties, carrageenan is used in wastewater treatment, particularly for removing cationic dyes, heavy metals, pharmaceuticals, and organic compounds such as phenol [98]. Carrageenan is also used in dentistry, cancer treatment, and biomedical engineering due to its biocompatibility, non-toxicity, and biodegradability in drug delivery systems, wound healing, and tissue regeneration [99].

Collectively, these marine biopolymers have attracted growing interest in environmental remediation technologies, particularly for the capture and immobilization of microplastics. Their natural origin, biodegradability, and the possibility of physicochemical modifications allow for the fabrication of functional materials such as gels, membranes, and nanocomposites. Moreover, chemical modification or the incorporation of inorganic components (e.g., metal nanoparticles or cross-linkers) has been shown to significantly enhance their sorption properties and their ability to participate in environmental remediation processes [39]. For example, due to their large specific surface area, functional group density, and porosity, chitosan-based nanocomposites and cellulose-based aerogels are highly effective at removing microplastics from seawater [100].

The use of plastics produced from petroleum poses a huge environmental problem. Therefore, bioplastics from natural and renewable biomass sources such as land plants, marine organisms, and microorganisms are being explored. Such bioplastics are biodegradable, compostable, and have a minimal carbon footprint. However, bioplastics produced from terrestrial plant sources (sugar cane, corn starch, banana and potato peels) account for a small percentage of annual plastic production due to their disadvantages, such as low strength, brittleness, and high production costs. Their production and biodegradability depend largely on atmospheric conditions. A promising alternative is bioplastics from marine sources—seaweed, microalgae, marine microorganisms, and marine animal waste. They are characterized by rapid growth and ease of cultivation. Bioplastics can be produced from many marine polysaccharides such as ulvan, cellulose, agar, carrageenan, alginate, starch, polylactic acid, chitosan, chitin, and collagen. Marine-based bioplastics are gaining increasing interest in the food, biomedical, agricultural, and industrial sectors [101,102].

According to market data for 2021 collected by European Bioplastics in collaboration with nova-Institute, bioplastic production is estimated to increase from 5 million tons in 2022 to over 7.5 million tons in 2026 (including 2.3 million tons of biobased non-biodegradable and 5.3 million tons of biodegradable bioplastics) [101].

## 5. Mechanisms of Interaction Between Microplastics and Marine Polysaccharides

Interactions between MPs and marine polysaccharides are mediated primarily by electrostatic attractions, hydrophobic forces, and hydrogen bonding. The specific physicochemical characteristics of both MPs and polysaccharides largely determine the type and strength of their interactions [103]. The interactions that take place between the molecules of biopolymers, such as chitosan and alginate, and the environment are mainly in the form of hydrogen bonds and non-covalent forces such as van der Waals forces, while ionic and hydrophobic interactions occur sporadically [104]. In addition, the effectiveness and selectivity of adsorption are influenced by environmental conditions, including salinity, pH, and natural organic matter [105].

### 5.1. Electrostatic and Hydrophobic Interactions

Electrostatic interactions occur predominantly between charged functional groups on polysaccharides and oppositely charged microplastic surfaces. Chitosan, possessing protonated amino groups (–NH_3_^+^) under acidic conditions, effectively binds negatively charged MPs. Alginate and carrageenan, containing carboxylate (–COO^−^) and sulfonate (–SO_3_^−^) groups, respectively, primarily bind positively charged MPs or their surface additives (Table 3) [39,59,60]. Another aspect worth noting is the pH of the aqueous environment, as it plays an important role in shaping the surface charges of both microplastics and adsorbent particles, thus influencing the strength and nature of electrostatic interactions between them. Microplastics typically exhibit an isoelectric point around pH 4, meaning that at higher pH values their surface becomes negatively charged and can interact electrostatically with positively charged adsorbent particles. It is worth noting that the typical pH of seawater is between 7.5 and 8.4, which favors just such negative surface charges of microplastics [106,107,108,109].

Microplastics typically have a high degree of hydrophobicity, which means that they tend to interact with other highly hydrophobic substances [106,110,111]. Hydrophobic domains present in both MPs and polysaccharides additionally contribute to the binding process through van der Waals interactions and hydrophobic attractions, further stabilizing MP–polysaccharide complexes [36,112,113].

### 5.2. Analytical Techniques for Studying Microplastic–Polysaccharide Interactions

To thoroughly characterize MP–polysaccharide interactions, various analytical techniques have been employed, each providing complementary information regarding chemical composition, surface properties, and morphological characteristics (Table 4).

These methods collectively enhance the understanding of how marine polysaccharides interact with microplastics, thereby guiding the optimization of materials designed for environmental remediation.

### 5.3. Interactions Between Marine Polysaccharides and Microplastics

The interplay between marine-derived polysaccharides and microplastics (MPs) has a substantial effect on the transport, distribution, and ecological behavior of MPs in aquatic environments. These interactions often modify key physicochemical characteristics of MPs—such as surface charge, hydrophobicity, particle aggregation, and density—which in turn influence their environmental mobility and bioavailability [57,115,116].

Among the most notable effects is the promotion of microplastic aggregation by anionic polysaccharides like alginate and carrageenan. These compounds can increase particle size and density, leading to enhanced sedimentation and reduced concentrations of MPs in the water column [14,117,118]. Studies have shown that sodium alginate adsorbs onto the microplastic surface, initiating an agglomeration process that leads to stabilization of the microplastic suspension through a steric hindrance mechanism and an increase in ionic strength [119]. This sedimentation may reduce exposure risks for pelagic species; however, it simultaneously raises concerns about increased accumulation of MPs in benthic zones, where they may pose chronic threats to bottom-dwelling organisms [103,120,121].

Marine polysaccharides exhibit adsorptive capacities that open up a wide range of possibilities for their use in marine microplastic removal technologies. Among these biopolymers, sodium alginate has attracted particular attention as it is increasingly used in environmental clean-up technologies due to its widespread availability and low cost. Its modification to increase sorption efficiency is a frequent subject of research. The functionality of polysaccharides can be tailored by, among other things, transforming them into colloidal materials with different morphologies, such as spherical or non-spherical nano- and microspheres, fibrous structures or thin films [92,122]. Due to their biocompatibility, biodegradability and favorable mechanical properties, alginates—particularly in the form of calcium salt—are widely used as adsorbent materials in water treatment [119,123,124,125]. One example is the development of a monolithic adsorbent in which sodium alginate acts as a matrix and sodium phosphate acts as an active ingredient to capture microplastics. Such a material shows high resistance to interfering ions, strong acids and bases, as well as humic acids [119,125]. Another example is the use of modified hydrogels, such as sodium alginate hydrogel enriched with polydopamine (PDA). Containing both hydrophilic and hydrophobic groups, polydopamine can interact with a variety of surfaces through electrostatic, hydrogen, hydrophobic bonds, π-π and covalent interactions. Modification of sodium alginate with PDA significantly enhances its ability to adsorb microplastics due to the intensification of interfacial interactions [121,126]. Among the known alginate-based technologies are also highly porous sodium alginate sponge materials with excellent mechanical properties, such as reproducible compression resistance. Furthermore, these sponges have high porosity (approximately 82%) and large pore surface area. Literature data shows that an alginate sponge in the form of a 0.5 × 0.5 × 0.5 cm cube immersed in 100 mL of simulated seawater (30% NaCl) with 1 mg/L MPs achieved microplastic removal efficiency exceeding 92% [127].

The use of marine-derived polysaccharides in microplastic water purification processes confirms the existence of significant interactions between polysaccharides and microplastics, which may also be relevant in terms of their interactions in the environment. Microplastics present in water have a high capacity to adsorb onto the surface of algae. For example, the marine microalgae Fucus vesiculosus adsorbs as much as 94.5% of fluorescent polystyrene microplastic pollution. Algae cells have thin microchannels that trap plastic particles with a diameter of about 20 μm inside the algae tissues. The best sorption coefficients are observed around the cut surface of microalgae because alginate is released from the cell walls at this point [128]. The adsorption of microplastics by marine algae slows down growth and reduces chlorophyll-a production. Microplastic particles block access to light and air, which limits algae photosynthesis. Algae consisting of anionic polysaccharides more effectively adsorb positively charged microplastic pollutants [129]. In summary, the surface charge of microplastics and the chemical nature of algae are key factors in the adsorption of microplastics by algae [128].

Another marine polysaccharide used in water treatment is chitosan, which has been used for years as a flocculant, gel-based adsorbent or membrane filtration component [114,130]. Chitosan, with its cationic nature under acidic conditions, exhibits strong affinity for negatively charged microplastics. Through ionic and hydrogen bonding, it can form stable MP–chitosan complexes, which not only reduce MP mobility but also facilitate removal through filtration and sedimentation [131,132]. Due to its biodegradability, non-toxicity, and abundance of functional groups (such as –NH_2_ and –OH), chitosan enables effective removal of pollutants through adsorption and photocatalysis. The increase in the efficiency of chitosan-cellulose composites and aerogels that remove microplastics from seawater was observed primarily due to the ability of chitosan to effectively promote the aggregation of plastic particles, showing higher activity under different pH and salinity conditions [30,100,113]. These properties make chitosan-based systems highly promising for water treatment applications and reduce dependence on fossil fuel-derived materials (as a bioplastic, it can replace petroleum), thus aligning with circular economy concepts.

Importantly, polysaccharide binding also influences the behavior of co-contaminants associated with microplastic surfaces. Adsorption and desorption dynamics of substances such as heavy metals and POPs can shift in response to polysaccharide interaction, potentially altering the toxicity profile and environmental persistence of these complexed materials [133].

When investigating the relationship between microplastics and marine polysaccharides, it is important to consider environmental conditions such as pH, salinity or water temperature. Many experiments, including but not limited to investigations of the effects of extracellular polymeric substances and marine polysaccharides on microplastics, have been conducted at room temperature, which deviates from actual ocean conditions. The representation of real conditions is an important aspect for reliable modelling of the behavior of microplastics in the marine environment [33,113,134,135,136].

Understanding the mechanisms and consequences of these interactions is critical not only for predicting microplastic fate but also for designing targeted and efficient remediation strategies.

The presence of marine polysaccharides, among others, plays an important role in the movement of microplastics in the oceans, influencing their distribution both in the water depth and in bottom sediments. The complexity of these interactions highlights the need for further research into the links between biological and physical processes in the marine environment [137].

## 6. Application in Bioremediation and Future Directions

Marine polysaccharides offer considerable potential as environmentally sustainable materials for microplastic removal. Their biodegradability, abundance, and structural versatility have positioned compounds such as alginate, chitosan, and carrageenan as valuable candidates for eco-friendly water purification technologies [37].

Recent efforts have focused on enhancing the performance of these materials through chemical functionalization, the integration of magnetic components, and cross-linking strategies. These modifications significantly improve adsorption efficiency and operational feasibility in remediation systems [24,55].

Alginate-based hydrogels are particularly effective in capturing MPs via entrapment and surface interaction mechanisms. Incorporation of magnetic particles has yielded alginate composites capable of high-capacity binding and easy recovery using external magnets, making them practical for large-scale implementation, primarily in the treatment of industrial wastewater of pharmaceutical origin or the production of semi-synthetic antibiotics [65]. Sodium alginate is a natural polysaccharide that can be modified to form stable films. It has active sites (carboxyl and hydroxyl groups) that can bind to various contaminants. The addition of magnetic nanoparticles (MNPs) imparts magnetic properties to the composites. Alginate-based films with magnetic nanoparticles (MNPs) can be useful as pollutant absorbers in marine environments or for removing heavy metals or organic pollutants from wastewater. The alginate coating protects the MNPs, and the magnetic properties allow for easy collection with a magnet after use. This creates a stable, reusable nanocomposite material suitable for environmental applications, including removing contaminants from water. After use, the entire alginate–MNP composite can be quickly separated from the water using an external magnet. Moreover, additives such as catalyst nanoparticles (gold or palladium), immobilized on the alginate-coated MNP core, enable the catalysis of enzymatic reactions that naturally occur during the decomposition of microplastics in the environment [30,138].

The adsorption efficiency of hybrid and composite MPs coatings is improved by combining natural polymers, synthetic polymers, inorganic compounds, or nanoparticles. Such systems overcome the disadvantages of single-component coatings, ensuring minimal environmental impact while maintaining adequate functionality. For example, electrospun PLA–alginate fibers increase mechanical stability and water permeability by combining synthetic durability with natural hydrophilicity. The addition of FeO_4_ nanoparticles enables magnetic recovery, while the addition of clay increases roughness and thus increases MPs adsorption [30].

Chitosan-derived materials, including functionalized hydrogels and surface-modified nanoparticles, demonstrate high sorption capacities—especially against anionic microplastics. Their regenerability and cost-efficiency further support their use in circular remediation models [60]. Nanocrystalline chitosan–cellulose composites are characterized by the presence of hydrogen bonds and van der Waals interactions thanks to the combination of the electrostatic bonding of chitosan with the large surface area of cellulose, which allows microplastics to be captured from various locations [30].

Carrageenan, though less studied in this context, is gaining attention for its selective binding of positively charged microplastic particles. Recent formulations of functionalized carrageenan-based films and gels have shown encouraging results, although optimization for industrial or field-scale applications remains a challenge [94].

Marine polysaccharides are effective in wastewater treatment. Chitosan is particularly important due to its prevalence, biodegradability, and ability to be modified to remove heavy metals, dyes, and other organic pollutants. The coagulation and flocculation properties of chitosan (resulting from its cationic charge) can be used to remove negatively charged colloidal organic or inorganic pollutants from wastewater. Since most colloidal pollutants are negatively charged, chitosan, with its cationic properties, is one of the most promising biopolymers for widespread use in wastewater treatment, and its coagulation effect is very effective compared to mineral coagulants such as aluminum sulfate, polyethyleneimine, and polyacrylamide, in removing various pollutants from aqueous solutions [139]. Furthermore, adsorption is known to be a reliable and economical alternative for removing pollutants from wastewater. Chitosan can be used as a biosorbent for heavy metal ions [140]. Alginate is also used as a catalyst in the removal of various pollutants from wastewater, such as metal ions or organic and inorganic pollutants. Through adsorption, alginate promotes the purification of wastewater from organic dyes, and after forming complexes with Cd, Cu, Au, Fe, Pb, Ni, or Zn, it removes heavy metals, pesticides, paints, and antibiotics [141]. Carrageenan effectively removes pollutants from wastewater by acting as a flocculant and adsorbent, and, in particular, removes heavy metals, dyes, and chemical compounds containing nitrogen and phosphorus. This is possible due to the negative charge of carrageenan and its ability to form complexes with pollutants. A major advantage of using carrageenan in wastewater treatment is its biodegradability, low toxicity, and ease of modification [142]. Microalgae participate in the adsorption of microplastics, achieving high sorption coefficients. For example, alginates contained in *Fucus vesiculosus* microalgae adsorb over 94% of polystyrene microplastic pollution [128]. *Pseudokirchneriella subcapitata* microalgae adsorb polystyrene particles with a diameter of 20–500 nm [129]. The physicochemical conditions, especially the temperature and pH of the water, have a significant impact on the adsorption of microplastics to the surface of microalgae. The charge on the surface of the particles has a significant impact on the sorption of microplastics on algae. Positively charged microplastic particles strongly adsorb to algae consisting of anionic marine-derived polysaccharides [129].

Continued research should focus on improving the long-term stability, selectivity, and reusability of these polysaccharide systems. Integration with other treatment technologies and comprehensive lifecycle analyses will be essential for their successful deployment in real-world environmental remediation. Future research should focus on optimizing the scalability, efficiency, and practical implementation of these polysaccharide-based bioremediation strategies. Interdisciplinary collaborations combining polymer chemistry, environmental engineering, biotechnology, and material science will be critical to addressing current limitations, such as material durability, economic feasibility, and potential ecological impacts of deploying these materials at large scales. Comparative efficiency of microplastic removal and modification methods were shown in Table 5 and Table 6.

## 7. Conclusions

The growing concern over microplastic (MP) contamination in aquatic environments highlights an urgent need for effective and sustainable mitigation strategies. Among the promising candidates for such solutions are marine-derived polysaccharides—namely alginate, chitosan, and carrageenan—valued for their natural origin, low environmental footprint, biodegradability, and economic feasibility.

These polysaccharides exhibit a strong capacity to interact with MPs through a combination of electrostatic forces, hydrophobic associations, hydrogen bonding, and physical entrapment. These interactions can significantly alter the physicochemical characteristics of microplastics, influencing their aggregation behavior, sedimentation rates, and transport pathways within aquatic systems. By promoting removal from the water column and reducing exposure to pelagic species, these biopolymers offer a dual environmental benefit: mitigating both physical microplastic stress and associated chemical pollutants, which are both absorbed and adsorbed to MPs.

Advances in material science have further expanded the potential of these biomaterials. Functionalization techniques—such as sulfonation, amination, cross-linking, or the integration of magnetic nanoparticles—have been shown to enhance the binding efficiency, structural stability, and recyclability of polysaccharide-based adsorbents. These improvements move the technology closer to practical deployment in large-scale water treatment and environmental remediation operations.

Despite these encouraging developments, several challenges persist. The environmental behavior and long-term fate of MP–polysaccharide complexes in real-world conditions remain poorly understood. Further research is needed to determine degradation pathways, interactions with natural organic matter and biota, and possible unintended effects in benthic ecosystems. In addition, systematic lifecycle assessments and cost–benefit analyses are critical to evaluate the broader sustainability of deploying these materials at scale.

Looking ahead, successful implementation of polysaccharide-based technologies will depend on coordinated efforts across multiple disciplines. Collaboration among chemists, environmental engineers, materials scientists, and ecotoxicologists is essential to address knowledge gaps and technical limitations. Policy and regulatory frameworks must also evolve to support the integration of biopolymer-based solutions into existing waste management and water purification infrastructures.

In summary, marine polysaccharides present a compelling, nature-based strategy for tackling the complex problem of microplastic pollution. Unlocking their full potential will require both continued scientific innovation and pragmatic planning to ensure these materials are not only effective in the lab but also reliable, safe, and scalable in diverse environmental contexts.

## Figures and Tables

**Table 1:** Microplastics derived from popular polymers (chemical structures prepared by the author).

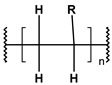	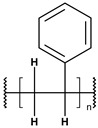		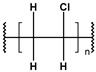
PE (R=H); PP (R=Me)	PS		PVC
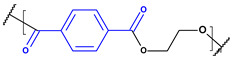	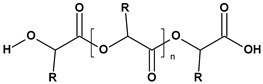		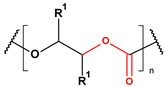
PET	PGA (R=H); PLA (R=Me)		PPC
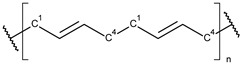		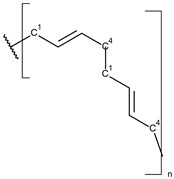	

PE—PolyEthylene; PET—PolyEthylene Terephthalate; PGA—Poly(Glycolic Acid); PLA—Poly(Lactic Acid); PP—PolyPropylene; PPC—PolyPropylene Carbonate; PS—PolyStyrene; PVC—PolyVinyl Chloride.

**Table 2:** Carbohydrates applied for removal of microplastic (chemical structures prepared by the author).

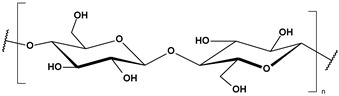	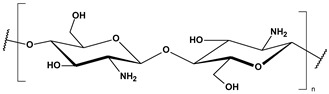
CEL	CTS
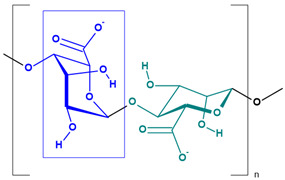	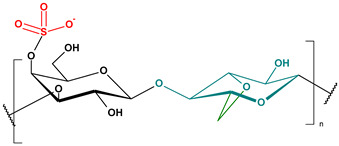
ALG (GM)	CGN

ALG—Alginate; CEL—Cellulose; CGN—Carrageenan; CTS—Chitosan.

**Table 3:** Comparative properties and microplastic binding mechanisms of major marine-derived polysaccharides.

Polysaccharide	Source	Main Functional Groups	Surface Charge	MPs Binding Mechanisms	Typical Applications	Ref.
Alginate	Brown algae	Carboxyl (–COO^−^)	Anionic	Electrostatic, gelation, surface adsorption	Hydrogels, water purification	[39,59]
Chitosan	Crustacean shells	Amino (–NH_3_^+^)	Cationic	Electrostatic, hydrogen bonding, hydrophobic	Magnetic filters, pollutant removal	[60,61,62,66]
Carrageenan	Red algae	Sulfonate (–SO_3_^−^)	Anionic	Electrostatic, ion complexation, hydrophobic	Adsorptive composites, bioremediation	[65,94]

**Table 4:** Analytical techniques for studying microplastic–polysaccharide interactions.

Technique	Purpose	Principle	Advantages	Limitations	Ref.
Fourier Transform Infrared (FTIR)	Polymer identification and chemical characterization	Molecular vibrations identification	Non-destructive	Low spatial resolution in case of testing very small samples, e.g., in micro scale	[31,55]
Raman Spectroscopy	Surface chemical analysis	Inelastic scattering of monochromatic light	High spatial resolution, non-destructive	Possible fluorescence interference	[18,32,64]
Scanning Electron Microscopy (SEM)	Surface morphology analysis	Electron beam imaging	High-resolution images	Requires vacuum	[57,114]
Atomic Force Microscopy (AFM)	Surface topography and force measurement	Surface scanning with mechanical probe	Nanoscale resolution	Small scan area, time-consuming	[59]
Zeta Potential	Surface charge determination	Electrophoretic mobility measurement	Quick, surface charge information	Sensitive to environmental conditions	[56,114]
Adsorption Isotherms	Quantification of adsorption capacity	Equilibrium adsorption measurements	Quantitative data on binding strength	Requires careful experimental setup	[61]

**Table 5:** Comparative efficiency of microplastic removal using marine polysaccharides and their modifications.

Material/Modification	Polysaccharide Type	Microplastic Type (Size)	Removal Efficiency (%)	Conditions	References
Chitosan-coated magnetic nanoparticles	Chitosan	PS (0.5–2 µm)	90–95	pH 5.5, 30 °C, 12 h	[56,60]
Alginate beads	Alginate	PE (1–5 µm)	75–85	pH 6.5, 25 °C, 24 h	[141]
Carrageenan hydrogel	Carrageenan	PP (2–10 µm)	65–80	pH 7.0, 20 °C, 48 h	[142]

**Table 6:** Modification methods of marine polysaccharides enhancing MPs removal.

Method	Polysaccharide	Effect on MPs Removal Efficiency	Application Example	References
Amino-functionalization	Chitosan	Improved selectivity for anionic MPs	Water purification systems	[60]
Cross-linking	Alginate, Chitosan	Increased mechanical stability, adsorption	Stable MPs capture gels	[139]
Sulfonation	Carrageenan	Enhanced cationic MPs binding	Adsorption composites	[140]
Nanoparticle addition	Alginate, Chitosan	Enhanced adsorption, magnetic recovery	Magnetic separable composites	[139]

## Data Availability

No new data were created or analyzed in this study.

## Abbreviations

The following abbreviations are used in this manuscript:

ALG	Alginic Acid
CEL	Cellulose
CTS	Chitosan
CGN	Carrageenan
MPs	Microplastics
NPs	Nanoplastics
PA	Polyamide
PDA	polydopamine
PE	Polyethylene
PET	Polyethylene terephthalate
PGA	Poly(glycolic acid)
PLA	Poly(lactic acid)
POPs	Persistent organic pollutants
PP	Polypropylene
PPC	Polypropylene carbonate
PS	Polystyrene
PVC	Polyvinyl Chloride
